# Editorial: Success and Failures in Implementing Health-Related Changes

**DOI:** 10.3389/fpsyg.2022.848049

**Published:** 2022-03-04

**Authors:** Magdalena Poraj-Weder, Irena Jelonkiewicz-Sterianos, Aneta Pasternak, Lidia Zabłocka-Żytka, Marja Kaunonen, Christophe Matthys, Alexander Mario Baldacchino, Jan Czesław Czabała

**Affiliations:** ^1^Institute of Psychology, The Maria Grzegorzewska University, Warsaw, Poland; ^2^Institute of Pedagogy and Psychology, Warsaw Management University, Warsaw, Poland; ^3^Department of Health Sciences, Faculty of Social Sciences, Tampere University, Tampere, Finland; ^4^Department of Chronic Diseases and Metabolism, Clinical and Experimental Endocrinology, KU Leuven, Leuven, Belgium; ^5^School of Medicine, University of St Andrews, St Andrews, United Kingdom

**Keywords:** health-related changes, pro- and anti-health behaviors, effectiveness of the health behavior change, health behavior outcomes, future directions in research and practice

The presented Research Topic is intended to provide a platform for showcasing the latest research on the psychological determinants and correlates of effectiveness in the health behavior change process in relation to both physical and mental health. Although, in recent years, a crisis in health promotion has been observed, especially in Western Europe (Woodall et al., 2018; Woodall and Freeman, 2020), the rise of civilization diseases affecting physical and mental health and the health crisis related to the COVID-19 pandemic (Abel and McQueen, 2020; Van den Broucke, 2020; Di Ruggiero and Ardiles, 2021) undoubtedly indicate the need for further research and recommendations regarding health psychology.

The Research Topic contains nine articles (eight research papers and one opinion) that address four areas: motivation to change (Poraj-Weder et al.; Rowicka), intrapersonal factors conducive to maintaining change (Burnos and Skrobowski; Mierzyńska et al.; Zawadzka et al.), the effectiveness of interventions (Iwon et al.; Skarin et al.), and the unhealthy behaviors and their determinants (Starosta et al.). Finally, due to the fact that, today, a key factor leading to changes in health behavior is the COVID-19 pandemic, this theme was also included (Dobrenko). The presented series of articles illustrates the diversity of Research Topics realized in the field of health psychology.

One important factor responsible for successes or failures in the process of changing health behavior is the quality of motivation to change (Poraj-Weder et al.; Rowicka). In the study of Poraj-Weder et al. an attempt was made to construct and validate a tool for measuring pro-health behavior motives, in the paradigm of Self-Determination Theory (Ryan and Deci, 2000, 2017). The problem of motivation to change in the specific context of recovery from alcohol addiction was addressed by Rowicka. In this study, two measurement tools were also adapted to assess the quality of motivation to change. Designing (Poraj-Weder et al.) and validating (Poraj-Weder et al., Rowicka) research tools does not only allow a better understanding of the underlying mechanisms of implementing health changes, but also a better selection of interventions. It is worth emphasizing that we are still lacking valid instruments to measure the effectiveness of health behavior change.

The unhealthy behaviors and their determinants are the subject of a study by Starosta et al. The authors engaged in finding the predictors of the problematic viewing of TV series by young adults. The results indicate a significant relationship between anxiety-depressive syndrome and the motivation to watch TV series and problematic series viewing. Several other articles (Burnos and Skrobowski; Mierzyńska et al.) focus on intrapersonal factors that facilitate change maintenance in a group of chronically somatically ill patients and in adolescent functioning (Zawadzka et al.). Patient engagement and adherence to medical recommendations is currently an important issue that has been addressed in many publications (Gu et al., 2021; Newman et al., 2021). Committing to health behaviors after heart transplantation is a crucial task in view of maintaining good health and the need to follow medical advice. Mierzyńska et al. concentrate on intrapersonal factors affecting health, such as personality traits, health behaviors, self-efficacy, and a health locus of control. Among the examined resources, the best predictors of caring about health were a health locus of control and the level of conscientiousness, according to Big Five concept. Among the various forms of health-relevant habits, maintaining a healthy diet proved to be the most difficult for heart transplant patients. In turn, in the group of patients with metabolic disorders (Burnos and Skrobowski), the moderating role of personality and temperamental traits was confirmed with regard to changes in health behavior and quality of life after the application of a motivational intervention. Higher Sensory Sensitivity, lower Perseveration, and higher Agreeableness enhanced positive change in health behaviors.

The importance of interpersonal factors in resisting negative environmental influences was investigated by Zawadzka et al. The authors analyzed the role of self-esteem, in a group of adolescents, in counteracting four sources of social materialism: that introduced by the mother, the father, peers, and the media. Ultimately, it was revealed that balanced self-esteem, as an element of mental health (Lehtinen, 2008), reduces the impact of materialism displayed by peers on the materialism of the adolescents in question.

Another problem addressed by the authors (Iwon et al.; Skarin et al.) was the effectiveness of interventions in the context of sustaining pro-health behavior. Skarin et al. used mixed-methods. Summarizing and integrating quantitative data (questionnaires and BMI measure) and qualitative data (pre-interviews and follow-up-interviews), they showed that the extension and maintenance of behavior change depends primarily on the subjective experience of goal achievement, combined with a reinforcing conversation before beginning the intervention.

The study by Iwon et al. examined changes in subjective well-being (happiness, life satisfaction, and self-esteem) in women who had just started engaging in, did engage in, or did not engage in physical activity. Upon re-measurement, an improvement in subjective well-being was found after 4 weeks in those who began physical activity.

The understanding of health was significantly influenced by the COVID-19 pandemic, provoking reflection on the need to make changes and take action to promote health (Duhigg, 2012; Ingram et al., 2020). Citing a number of research findings and her own experience as a psychotherapist and a psychologist supporting personality development, Dobrenko describes the process of forming attitudes that promoinge healthy behavior and stresses its automatic nature. According to Dobrenko, it is advisable to choose new habits based on the needs that are currently inadequately met, including affiliation, stimulation, activity, and rhythm. These newly developed healthy habits can work to our long-term advantage once they become automatic, which requires finding a cue that will trigger a habit, developing a routine that can be habitually followed, and defining the reward).

The collected papers, addressing change in health and disease, clearly demonstrate that health promotion should be committed to both salutogenesis and pathogenesis (Woodall and Freeman, 2020). The distinction between health promotion and disease prevention (Tengland, 2010) seems artificial in the context of contemporary challenges and emerging disease outbreaks (Laverack, 2017), including COVID-19. Because implementing sustainable change is difficult, research must identify the determinants of effective intervention (Conner and Norman, 2017). There is also a need for methods to identify the motivational status of the person who undertakes change so that it can be effective and, above all, sustainable (Poraj-Weder et al., 2021). Carrying out research in a mixed-method approach seems very promising in this context.

The analysis of the collected material indicates the usefulness of models integrating both conscious, reflexive processes and unconscious, automatic ones. Research taking into account the influence of motivational (e.g., behavioral intention), volitional (e.g., planning) and automatic (e.g., habits) processes on health behaviors allows us to better predict the intention to undertake health behaviors and regulatory strategies that will actually work (Arnautovska et al., 2017).

We hope that the articles presented in the issue “**Success and Failures in Implementing Health-Related Changes**” will inspire further research and reflection in the area of health psychology and health behavior change and maintenance, as well as effective interventions in this area.

## Author Contributions

All authors listed have made a substantial, direct, and intellectual contribution to the work and approved it for publication.

## Conflict of Interest

The authors declare that the research was conducted in the absence of any commercial or financial relationships that could be construed as a potential conflict of interest.

## Publisher's Note

All claims expressed in this article are solely those of the authors and do not necessarily represent those of their affiliated organizations, or those of the publisher, the editors and the reviewers. Any product that may be evaluated in this article, or claim that may be made by its manufacturer, is not guaranteed or endorsed by the publisher.
